# Quantum Information Entropies on Hyperbolic Single Potential Wells

**DOI:** 10.3390/e24050604

**Published:** 2022-04-26

**Authors:** Carlos Ariel Gil-Barrera, Raymundo Santana Carrillo, Guo-Hua Sun, Shi-Hai Dong

**Affiliations:** 1 Centro de Investigación en Computación (CIC), Instituto Politécnico Nacional, UPALM, Ciudad de México C.P. 07700, Mexico; cgilb2020@cic.ipn.mx (C.A.G.-B.); a200307@sagitario.cic.ipn.mx (R.S.C.); gsun@cic.ipn.mx (G.-H.S.); 2 Research Center for Quantum Physics, Huzhou University, Huzhou 313000, China; 3 Laboratorio de Información Cuántica, CIDETEC, Instituto Politécnico Nacional, UPALM, Ciudad de México C.P. 07700, Mexico

**Keywords:** hyperbolic potential well, quantum information entropy, position and momentum Shannon entropies, 03.65.–w, 03.65.Ge, 03.67.–a

## Abstract

In this work, we study the quantum information entropies for two different types of hyperbolic single potential wells. We first study the behaviors of the moving particle subject to two different hyperbolic potential wells through focusing on their wave functions. The shapes of these hyperbolic potentials are similar, but we notice that their momentum entropy densities change along with the width of each potential and the magnitude of position entropy density decreases when the momentum entropy magnitude increases. On the other hand, we illustrate the behaviors of their position and momentum entropy densities. Finally, we show the variation of position and momentum entropies $S_{x}$ and $S_{p}$ with the change of the potential well depth *u* and verify that their sum still satisfies the BBM inequality relation.

## 1. Introduction

Since quantum mechanics was founded, one of its fundamental tasks is to explore the exact solutions of the nonrelativistic equation for different kinds of potential fields [1,2,3]. This is because the exact solutions include all quantum information of studied quantum systems required for understanding the fundamental features. One of its important applications is related to the Shannon entropy when scientists such as Beckner, Bialynicki-Birula and Mycielski (BBM) obtained an entropic uncertainty relation [4], which is given by

(1) $$S_{x}+S_{p}\geq D(1+\ln\pi )$$

where *D* denotes the spatial dimension.

Up to now, Shannon theory has gained importance in different areas such as quantum computing, quantum cryptography and others [5,6,7,8] due to its basis for data transmission, signal processing, and data measurement [9,10,11]. On the other hand, the Shannon entropy also reflects the localization information of the probability distribution. Until now, many works have focused on calculating the information entropy for soluble quantum systems, e.g., the Rosen-Morse Potential [12], the double-well potential [13], the Pöschl-Teller like potential [14] or infinite circle well [15]. Particularly, they also include basic and interesting systems such as the harmonic oscillator [16], quantum dots [17] and the finite single well [18]. Moreover, the Shannon information entropy has also become an important interdisciplinary subject representing a universal concept in statistical physics [19,20].

However, we should recognize that it is not easy to calculate exactly Shannon entropy except for a few soluble systems mentioned above [11,12,13,14,15,16,17,18]. Most of them have to be computed numerically. Although the present study can be expressed explicitly by the confluent Heun function [21], unfortunately, the calculation of the wave function in momentum space becomes a challenging task since the Fourier transform involving the confluent Heun function cannot be performed easily. Therefore, we must deal with this problem in a numerical way. This shall enrich and extend our recent study of the asymmetric multiple quantum wells with a constant total length [22,23,24].

This paper is organized as follows. In Section 2, we present the formalism to this system subject to hyperbolic single well potentials. In Section 3, we first present the position and momentum entropy densities and then calculate the position $S_{x}$ and momentum $S_{p}$ for lower-lying states. The key point is about the wave function in momentum space, which is calculated via the Fast Fourier transform (FFT) approach. Finally, we give concluding remarks in Section 4.

## 2. Formalism

Generally, the one-dimensional Schrödinger equation for a nonrelativistic particle of mass M subject to a hyperbolic potential can be written as:

$$-\frac{\hbar^{2}}{2M}\frac{d^{2}\psi \left(x\right)}{dx^{2}}+U_{q}\left(x;u,L\right)\psi \left(x\right)=E\psi \left(x\right)$$

with

(2) $$U_{q}(x;u,L)=-\frac{\hbar^{2}u}{2M\;L^{2}}\frac{1}{\cosh^{4}(x/L)}\;,\;-\frac{\hbar^{2}u}{2M\;L^{2}}\left[\frac{\;\cosh(2x/L)}{\cosh^{4}(x/L)}\right],\;q\in [1,3],$$

where the parameters u and L denote the depth and width of the potential wells $U_{q}\left(x;u;L\right)$, respectively. For simplicity and convenience, we take L=1 and same notations *U*_1,3_ as defined in [21]. Thus, Equation (1) can be transformed to the following form:

(3) $$-\frac{d^{2}\psi (x)}{dx^{2}}+U_{q}(x;u)\;\psi (x)=\varepsilon \;\psi (x),$$

where

(4) $$U_{q}(x;u)=-\frac{u}{\cosh^{4}(x)}\;,\;-\frac{\;u\cosh(2x)}{\cosh^{4}(x)},\varepsilon =\frac{2ME}{\hbar^{2}}<0.$$

**Figure 1:**
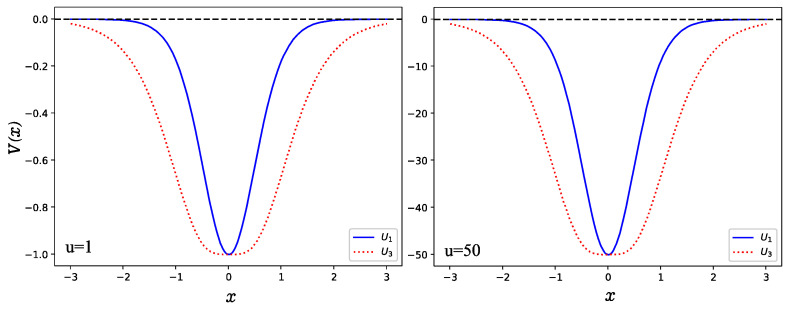
Hyperbolic potential with two different potential well depths u=1 and u=50, respectively.

**Figure 2:**
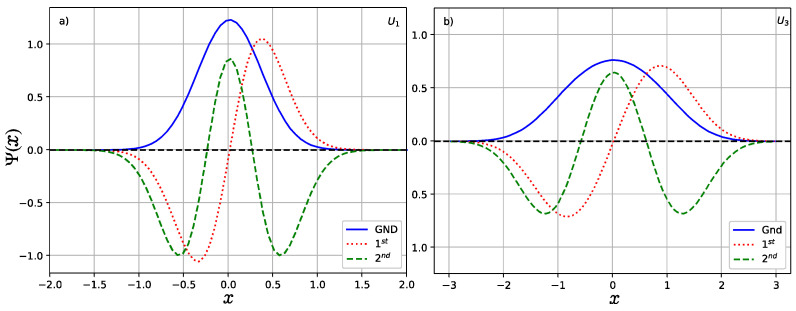
Wave functions of lower-lying states (**a**) for $U_{1}$ and (**b**) for $U_{3}$.

As shown in Figure 1, the number of bound states is finite for all $U_{q}$ for a given potential parameter *u* and both $U_{1,3}$ are a single well with even parity and maximum depth u. In Figure 2, we show the wave functions of lower-lying states which satisfy relation $\psi_{n}\left(-x\right)={\left(-1\right)}^{n}\psi_{n}\left(x\right)$, where n denotes the number of nodes in the wave function. In the study, we take the potential parameter u=1. It is shown that the amplitude of the wave function in *U*_1_ is greater than that of the case *U*_3_ but the interval of the moving particle in the case *U*_1_ is narrower than that of the case *U*_3._ This can be explained well by their potential shapes as shown in Figure 1, in which the width for *U*_3_ (red dotted line) is larger than that of the *U*_1_ (blue solid line).

Let us study the Shannon entropy, which includes two parts, i.e., the position and momentum entropies defined by $S_{x}$ and $S_{p}$, respectively. The Shannon information entropy densities *ρ*_s_(*x*) and *ρ*_s_(*p*) are defined as [12,13,14,15],

(5) $$\rho_{s}(x)={\left|\psi (x)\right|}^{2}\ln({\left|\psi (x)\right|}^{2}),\;\rho_{s}(p)={\left|\varphi (p)\right|}^{2}\ln({\left|\varphi (p)\right|}^{2})$$

where the notations ${\left|\psi_{n}\left(x\right)\right|}^{2}$ and ${\left|\varphi_{n}\left(p\right)\right|}^{2}$ represent their probability densities in position and momentum space. The wave function *φ*(*p*) can be calculated by using the Fourier transform

(6) $$\varphi (p)=\frac{1}{\sqrt{2\pi }}\int \psi (x)e^{-i\;p\;x}dx$$

Based on Equation (5), the position and momentum space information Shannon entropies $S_{x}$ and $S_{p}$ are defined, respectively, as

(7) $$S_{x}=-\int_{-\infty }^{+\infty }\rho_{s}(x)dx,\;S_{p}=-\int_{-\infty }^{+\infty }\rho_{s}(p)dp$$

Among the existing reference books, there is no book that explains how to obtain such a transformation containing the confluent Heun function. This leads to the fact that the Fourier transform involving the confluent Heun function cannot be performed easily. Therefore, we computed the position and momentum entropies using the numerical method. To this end, we applied FFT to compute the normalized wave function in momentum space, except for the wave function in position space.

## 3. Results and Discussions

**Figure 3:**
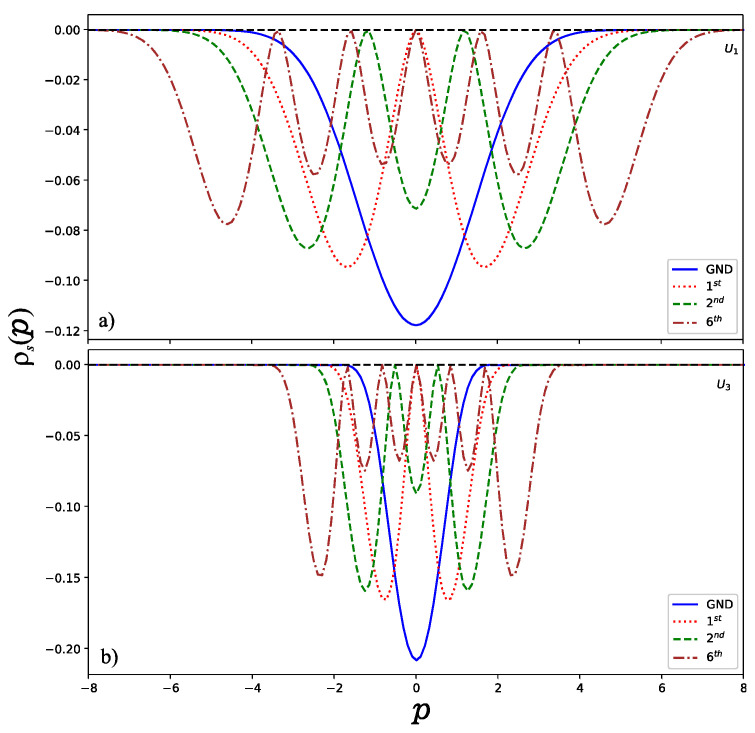
Momentum entropy densities (**a**) for $U_{1}$ and (**b**) for $U_{3}$.

**Figure 4:**
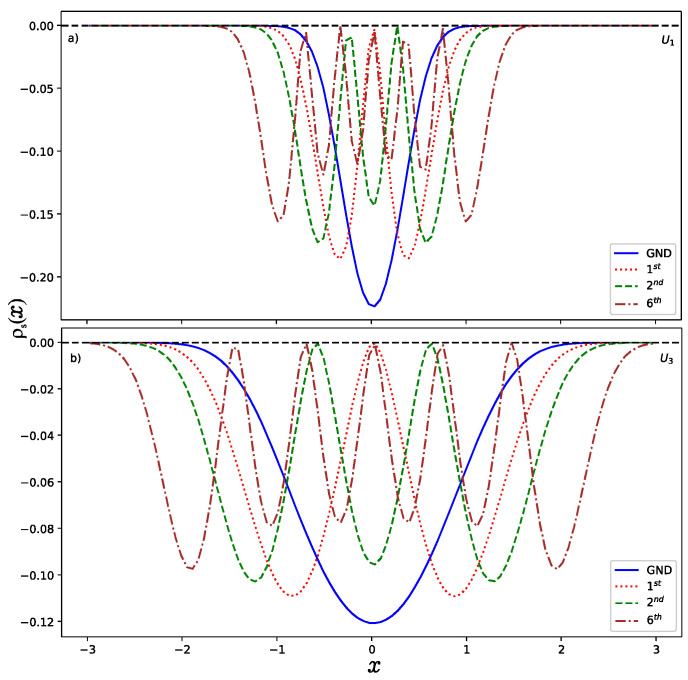
Position entropy densities (**a**) for $U_{1}$ and (**b**) for $U_{3}$.

Now, let us present the results obtained in this work and remark on some particularities. Since this work focused on a numerical solution of the Schrödinger equation, we created a program in Python to compute the normalized wave functions as a function of the variable x. Once we had the wave function, then it was possible to obtain the wave function in momentum space via the FFT method. In Figure 3 and Figure 4, we show the momentum and position entropy densities for the ground, first, second and sixth states of each hyperbolic potential. It is found that the width of the momentum entropy density for $U_{1}$ is wider than that of $U_{3}$, but their widths of the position entropy density are converse. This is also related to their potential shapes.

**Figure 5:**
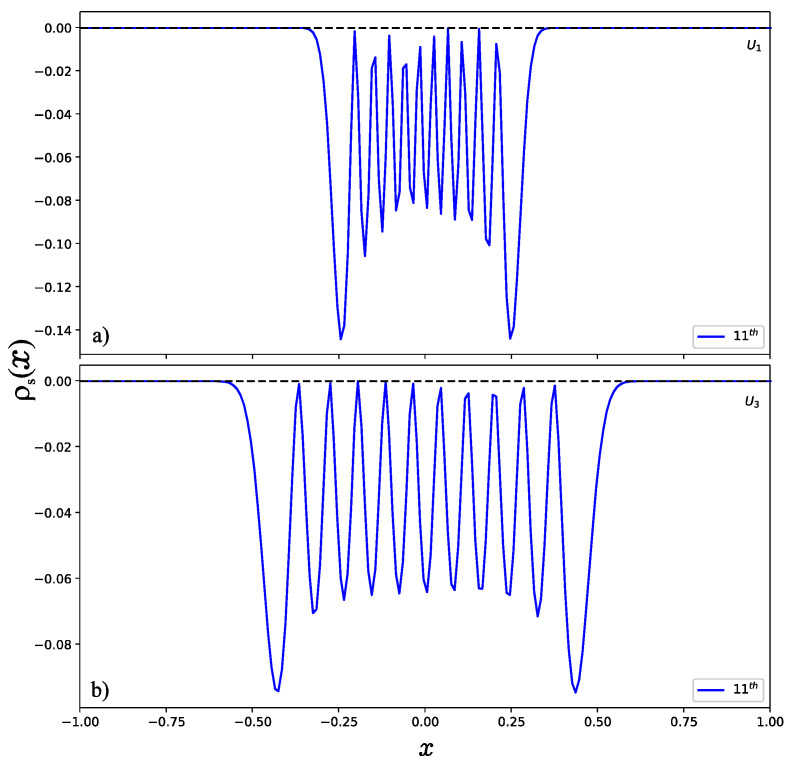
Position entropy density of the normalized 11th excited state (**a**) for $U_{1}$ and (**b**) for $U_{3}$.

**Figure 6:**
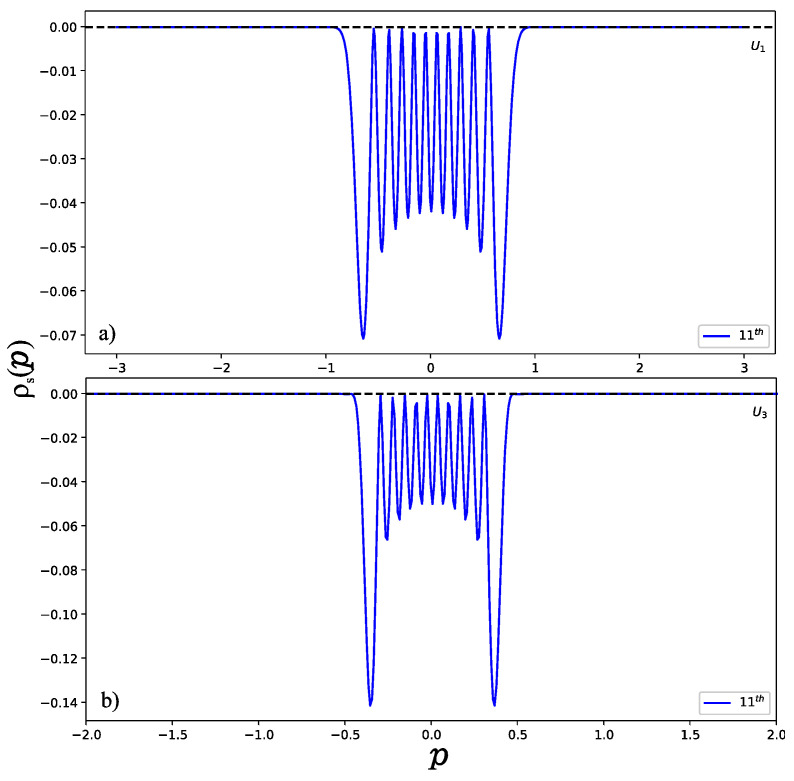
Momentum entropy density of normalized 11th excited state (**a**) for $U_{1}$ and (**b**) for $U_{3}$. For $U_{1}$ shown in (**a**), we notice that the effective range for the momentum entropy density on the 11th excited state is near to (−1, 1), while for $U_{3}$ in (**b**), it is near to (−0.5, 0.5).

To illustrate the behavior of the wave function in higher excited states, we have to take a large value of the potential parameter *u*. Otherwise, the system does not support enough bound states if the potential parameter *u* is very small. To observe the position entropy density of the 11th normalized excited state (*u* = 300) as shown in Figure 5, we found that the position entropy density is mainly distributed in the interval (−0.25,0.25) for $U_{1}$ as shown in Figure 5a, but in the interval (−0.5, 0.5) for $U_{3}$. This can be explained well by the shapes of the potential wells as shown in Figure 1, since the width of $U_{3}$ is wider than that of $U_{1}$. However, the momentum entropy density is contrary to that of the position entropy density. As displayed in Figure 6, for $U_{1}$ shown in Figure 6a, it is possible to see that the effective range for the momentum entropy density on the 11th excited state is near to (−1, 1), while for $U_{3}$ in Figure 6b, it is near to (−0.5, 0.5).

**Figure 7:**
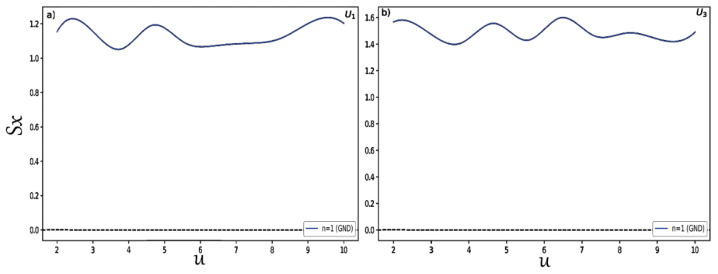
Position entropy $S_{x}$ for distinct depths (**a**) for $U_{1}$ and (**b**) for $U_{3}$.

**Figure 8:**
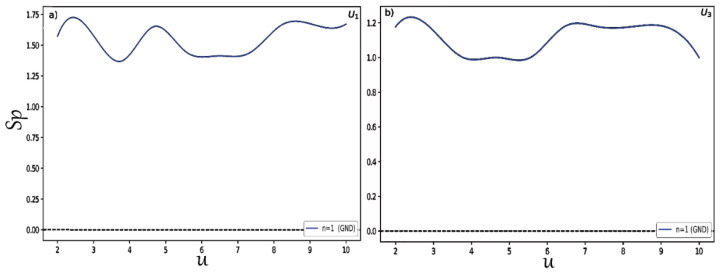
Momentum entropy $S_{p}$ for distinct depths (**a**) for $U_{1}$ and (**b**) for $U_{3}$.

**Figure 9:**
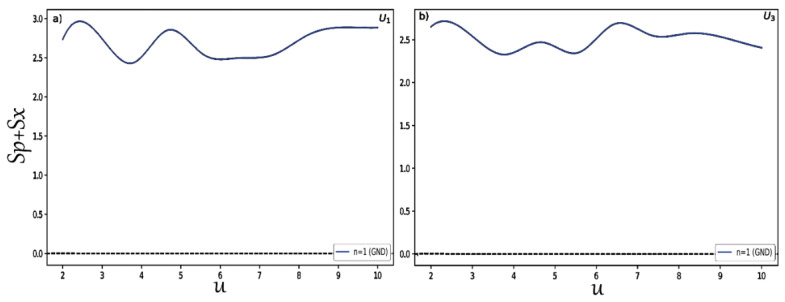
Sum of position $S_{x}$ and momentum $S_{p}$ entropies in the hyperbolic single quantum wells as a function of the depth *u*. Panel (**a**) is for the case $U_{1}$, while (**b**) is for $U_{3}$.

Finally, to study in detail the behavior of the position and momentum entropies in hyperbolic potentials and examine that the BBM inequality relation is satisfied in these two systems, we changed the depth u for potentials $U_{1,3}$ and we plotted the results shown in Figure 7 for the position entropy $S_{x}$. In the same way, we present in Figure 8 the variation of the momentum entropy $S_{p}$ with respect to the depth u of the potential wells. Here, we only considered the normalized ground states for each type of potential. It is clearly shown that the position and momentum entropies do not always increase and decrease for some depths. It is also found that the BBM inequality relation is always satisfied, as shown in Figure 9. That is, their sum always stays above the value 2.1447.

## 4. Concluding Remarks

In this work, we investigated the position $S_{x}$ and momentum $S_{p}$ Shannon information entropies for the quantum system subject to hyperbolic potential single wells. We solved this system numerically and illustrated the position and momentum entropy densities as well as the Shannon entropies. Furthermore, we found that the BBM inequality is satisfied for the position $S_{x}$ and momentum $S_{p}$ even when the potential depth changes or when these magnitudes increase or decrease. We consider that it is worthwhile to study hyperbolic potential wells, possibly with different variations, e.g., a double potential well; we are working on this research.

## Data Availability

The study did not report any data.
